# Production of a Functional Frozen Yogurt Fortified with* Bifidobacterium* spp.

**DOI:** 10.1155/2017/6438528

**Published:** 2017-06-11

**Authors:** Amro Abdelazez, Zafarullah Muhammad, Qiu-Xue Zhang, Zong-Tao Zhu, Heba Abdelmotaal, Rokayya Sami, Xiang-Chen Meng

**Affiliations:** ^1^Key Laboratory of Dairy Science of Ministry of Education, Northeast Agricultural University, Harbin 150030, China; ^2^Department of Dairy Microbiology, Animal Production Research Institute, Agriculture Research Center, Dokki, Giza 12618, Egypt; ^3^Department of Microbiology, Soil, Water and Environment Research Institute, Agriculture Research Center, Giza 12619, Egypt; ^4^Department of Microbiology and Biotechnology, College of Life Sciences, Northeast Agricultural University, Harbin 150030, China; ^5^Department of Nutrition and Food Science, Taif University, Taif, Al-Huwayah 888, Saudi Arabia; ^6^Department of Food Science, Northeast Agricultural University, Harbin, Heilongjiang 150030, China

## Abstract

Frozen dairy products have characteristics of both yogurt and ice cream and could be the persuasive carriers of probiotics. Functions of the frozen yogurt containing viable bifidobacterial cells are recognized and favored by the people of all ages. We developed a kind of yogurt supplemented by* Bifidobacterium* species. Firstly, five strains of* Bifidobacterium* spp. (*Bifidobacterium bifidum *ATCC 11547,* Bifidobacterium longum* ATCC 11549,* Bifidobacterium infantis *ATCC 11551,* Bifidobacterium adolescentis *ATCC 11550, and* Bifidobacterium breve* ATCC 11548) were evaluated based on the feasibility criteria of probiotics, comprising acid production, bile tolerance, and adhesion to epithelial cells. Formerly, we combined the optimum strains with yogurt culture (*Lactobacillus delbrueckii* subsp.* bulgaricus* EMCC 11102 and* Streptococcus salivarius* subsp.* thermophilus* EMCC 11044) for producing frozen yogurt. Finally, physiochemical properties and sensory evaluation of the frozen yogurt were investigated during storage of 60 days at −18°C. Results directed that* Bifidobacterium adolescentis *ATCC 11550 and* Bifidobacterium infantis *ATCC 11551 could be utilized with yogurt culture for producing frozen yogurt. Moreover, the frozen yogurt fermented by two bifidobacterial strains and yogurt culture gained the high evaluation in the physiochemical properties and sensory evaluation. In summary, our results revealed that there was no significant difference between frozen yogurt fermented by* Bifidobacterium* spp. and yogurt culture and that fermented by yogurt culture only.

## 1. Introduction 

Diet plays an important role in preventing diseases and ensuring health. Hence, the consumption of functional foods (i.e., beneficial compounds or foods containing microorganisms) which provide health benefits with a reduction of coronary heart disease, obesity risk, and diabetes has increased during the last decade [1]. The concept of using probiotics to improve and maintain human health is not new at all. Probiotic microorganisms are usually used as culture concentrates in dried or deep-freeze forms to be added to food for industrial or home uses [2]. In addition to the probiotic foods, there are various health products and pharmaceutical preparations containing probiotics on the market [3].

*Bifidobacterium* is an important group of probiotic cultures and commonly used in fermented dairy products that contributes a major part in the human intestinal microbiota in healthy humans. They are considered to provide many beneficial effects including improvement of lactose digestibility, anticarcinogenic activity, reduction of serum cholesterol level, synthesis of B vitamins, and facilitation in calcium absorption [4]. Moreover, numerous studies with different strains of* Lactobacillus* and* Bifidobacterium* have been performed in vitro and in vivo, in humans and animal models to investigate their immunomodulatory properties and probiotic potential to treat various infectious, allergic, and inflammatory conditions [5, 6]. Even though* Bifidobacterium* strains have already been used in dairy products, they have some inferior behavioral characteristics compared with the traditional lactic acid bacteria (LAB) used in fermented dairy products, hindering their possible applications [7]. Vitally, they represent weaker growth and acid production in cow milk and require long fermentation times, anaerobic conditions, and low redox potential for their growth [8].

There are clear relationship between the food we eat and our health. Therefore, some reports have investigated ice cream and yogurt as probiotic carrier. Hence, frozen yogurt is a novel way of combining the characteristics of ice cream with the therapeutic properties of yogurt that are considered as a healthy alternative to ice cream for the people suffering from cardiovascular diseases and lactose intolerance [1, 9–12]. The aim of study was to examine different factors affecting survival and activity of five species of bifidobacteria, study the viability of two chosen* Bifidobacterium* species in manufactured frozen yogurt under different conditions, and investigate the effect of storage temperatures on their viability.

## 2. Materials

### 2.1. Additives

Skim milk powder, vanilla, and sugar were purchased from local market. Stabilizer, emulsifier, and Cremondan SE 38 veg were provided by Danisco Ingredients, Denmark.

### 2.2. Bacterial Strains

Freeze dried* Lactobacillus delbrueckii* subsp.* bulgaricus* EMCC 11102 and* Streptococcus salivarius* subsp.* thermophilus* EMCC 11044 and* Bifidobacterium* species including* Bifidobacterium bifidum* ATCC 11547,* Bifidobacterium longum* ATCC 11549,* Bifidobacterium infantis* ATCC 11551,* Bifidobacterium adolescentis* ATCC 11550, and* Bifidobacterium breve* ATCC 11548 were provided by Cairo Microbiological Resources Center, Egypt.

## 3. Methods

### 3.1. Determination of Maximum Growth Rate and Maximum Acidification of* Bifidobacterium* spp. Strains in MRSL

*Bifidobacterium* spp. were inoculated (1% v/v) and grown in MRSL (Man Rogosa Sharpe) broth (Oxoid, Basingstoke, UK) supplemented with 5% (w/v) lactose (Win Lab, Gemini House, Middlesex, Hab 7ET, UK) and 0.05% (w/v) L-cysteine-HCL (Merck, Germany) at 37°C under anaerobic conditions (BBL Gas Pak, Becton Dickinson, Cockeysville MA, USA). The bacterial growth was monitored by measuring the absorbance with a spectrophotometer (DU 800, Beckman Coulter, USA) at 660 nm. Moreover, pH was determined by using pH meter (MP 220, Metler Toledo, Greifensee, Switzerland). The maximum acidification rate was reported according to [13].

### 3.2. Bile Salts Tolerance of* Bifidobacterium* spp. 

According to [14]* Bifidobacterium* spp. strains were inoculated in MRSL broth added to 0.3% (w/v) oxgall powder (Merck, Germany) and incubated at 37°C under anaerobic conditions for 24 hr. Bacterial growth was monitored by measuring absorbance with a spectrophotometer at 660 nm after 24 hr. The obtained absorbance values were plotted against the incubation time. Strain inoculated in MRSL broth without oxgall powder was taken as the control. Correlation between all the results of* Bifidobacterium* spp. resistance to bile salts was determined by the principal component analysis (PCA) using XLSTAT software.

### 3.3. Calculation of Survival Rate in Bile Salts

The survival rate was calculated by using the following formula reported by [15]:

(1) $$\begin{array}{c} \%\;\;Bile\;\;survival=\frac{\log N_{1}}{\log N_{0}}\times 100. \end{array}$$

 
log⁡*N*_1_ is absorbance of culture in MRSL broth containing 0.3% bile salts.
 
log⁡*N*_0_ is absorbance of culture in MRSL broth without bile salts.

### 3.4. Adhesion of* Bifidobacterium* spp. to Intestinal Epithelial Cells

According to [13] for the adherence assay, five* Bifidobacterium *spp. strains were tested for the adherence to epithelial cells.* Bifidobacterium* spp. strains were inoculated in MRSL broth and incubated overnight at 37°C under anaerobic conditions. The cultures were adjusted overnight to 1.5 × 10^8^ CFU/ml and then 10 ml of* Bifidobacterium* spp. cultures was removed and centrifuged at 4000 ×g RPM for 12 min. The supernatant was discarded, and 10 ml PBS (pH 7.2) was added and mixed using vortex. The crop scraping of epithelial cells was prepared by scrapping off the epithelium from rabbit duodenum with the edge of a microscope slide, washed by phosphate buffered saline, and suspended in buffer (pH 7.2). Moreover, cell cultures were washed five times with sterile phosphate buffered saline (PBS) (pH 7.2). Thereafter, 0.4 ml of epithelial cell suspension was added to 0.1 ml of bacterial cell suspension. The mixture was centrifuged at 4000 ×g RPM for 5 min and then incubated at 37°C for 30 min. Finally, binding between the bifidobacterial cells and epithelial cells was examined by gram stained phase contrast microscopy (magnification fold, 200x). The adhered bifidobacterial cells were determined by counting adhering bifidobacterial cells in 15 randomly selected microscopic fields.

### 3.5. Manufacturing Procedure of Frozen Yogurt

#### 3.5.1. Preparation of Yogurt

Experimental plain yogurt was prepared by heating pasteurized whole milk at 72°C for 10 minutes and subsequently cooled to 43°C Then, it was divided into five separate containers:

*Formula 1 (C)* inoculated with 1% w/w starter yogurt culture with no* Bifidobacterium* spp.

*Formula 2 (C + A)* inoculated with 1% w/w starter yogurt culture + 1% w/w of* B. adolescentis. *

*Formula 3 (C + B)* inoculated with 1% w/w starter yogurt culture + 1% w/w of* B. infantis*.

*Formula 4 (A + B)* inoculated with 1% w/w* B. adolescentis *+ 1% w/w of* B. infantis*.

*Formula 5 (C + A + B)* inoculated with 1% w/w starter yogurt culture + 1% w/w* B. adolescentis *+ 1% w/w of* B. infantis.*

The inoculated mixtures were incubated at 37°C until the pH 5.9 was obtained.

### 3.6. Preparation of Frozen Yogurt

Five frozen yogurt blends, each of three replicates, were prepared. All mixtures were standardized to contain 8% fat, 12% milk solids not fat, 16% sugar, 0.8% stabilizer/emulsifier, and 0.3% vanilla. In each treatment, mixed ingredients were homogenized together by using the method described by [16] with some modifications and then heated at 80°C for 30 min. All mixes were cooled at 5°C and then aged overnight at the same temperature. On the other hand, prepared yogurt was added (10% v/v) to five ice cream mixes prior to freezing. The freezing was performed in a horizontal batch freezer (Taylor Co., USA) and hardened at −18°C for 24 h before analyses.

### 3.7. Physicochemical Analyses

Frozen yogurt samples were stored at −18 ± 2°C for 60 days, and the physicochemical analyses were performed at 0, 15, 30, and 60 d. Titratable acid (TA) and total solid (TS) were analyzed for all frozen yogurt samples according to [17], and pH was determined by pH meter (MP 220, Metler Toledo, Greifensee, Switzerland).

### 3.8. Overrun and Meltdown Tests

The overrun was calculated according to [17].

(2) $$\begin{array}{c} Overrun=\left[\;\frac{\left(W1-W2\right)}{W2}\;\right]\times 100, \end{array}$$

where *W*1 is weight of the mix and *W*2 is weight of the same volume of frozen yogurt. The meltdown test was conducted in a chamber with controlled temperature (25°C). According to the method described by [18]. Results were expressed as a time for collection of each 10 ml of liquid.

### 3.9. Hardness

Texture analysis was performed using Texture Analyzer (TA.XT Plus Texture Analyzer, UK). The samples were stored in 50 mm plastic containers at −18°C until analysis. Measurement was carried out by using a cylindrical probe. Penetration depth at the geometrical center of the sample was 10 mm and penetration speed was set at 2 mm/s. The hardness was determined as the peak compression force (g) during penetration [19].

### 3.10. Enumeration of Viable* Bifidobacterium* spp. in Frozen Yogurt

The viable bifidobacterial cell count in frozen yogurt samples containing* Bifidobacterium *spp. was determined and expressed as colony forming units (CFU/mL) during storage of 0, 15, 30, and 60 d at −18 ± 2°C. Bifidobacterial cell counts were enumerated on MRSL agar using pour plate technique. The plates were incubated anaerobically at 37°C for 72 hr. Survival rates percentage of bifidobacteria was calculated according to [20].

### 3.11. Sensory Appraisal

Organoleptic properties of frozen yogurt were evaluated after 60 days of storage according to [21], for flavor (45 points), body and texture (35 points), appearance (10 points), melting quality (10 points), and total scores (100 points) by 20 panelists of the experienced staff members of the Dairy Science Department, Faculty of Agriculture, Minia University, Egypt.

### 3.12. Statistical Analysis

All experiments and analyses were performed in triplicate. The results were given as means ± the standard error of mean (SEM) and analyzed by using Graph Pad Prism 5 software. Comparisons between groups were performed by using one-way analysis of variance (ANOVA) after *t*-test. In addition, *p* < 0.05 was considered significant. The PCA using XLSTAT software determined the correlation between all the experiments.

## 4. Results and Discussions

### 4.1. Growth Rate and pH of* Bifidobacterium* spp. in MRSL at 37°C

**Figure 1:**
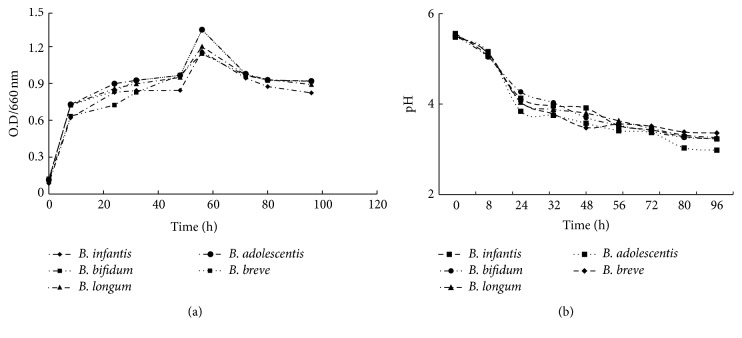
(a) Growth rate of different species of* Bifidobacterium *spp. (b) pH of different species of* Bifidobacterium *spp.

All the bifidobacterial species showed a similar growth profile when* Bifidobacterium* spp. were incubated in MRSL at 37°C. The first log phase was observed during the first 12 to 24 hr of growth and second log phase was started at 48 hr and continued until 56 hr and after that decline phase was started (Figure 1(a)).

The kinetics growth of five* Bifidobacterium* spp. and pH investigated that* B. adolescentis, B. breve,* and* B. longum* grown well in lactose MRS and the rates of growth were 1.363, 1.362, and 1.223 at 65 hr, respectively, at log phase, while results in Figure 1(b) have shown the decrease of pH gradually from 5.48 at zero time to 3.41, 3.56, and 3.63, respectively, after 65 hr. However, growth of the* B. adolescentis, B. breve, and B. longum *was 0.937, 0.935, and 0.907 at 96 hr, respectively, whereas pH was 2.98, 3.36, and 3.26 at 96 hr, respectively. On the contrary, growth of the* B. infantis and B. bifidum *was 1.183 and 1.164 at 65 hr of incubation and pH was 3.52 and 3.53, respectively. Meanwhile, the growth was 0.839 and 0.935 and pH 3.23 and 3.24, respectively, at 96 hr. These results were in complete consensuses with [13] that have attributed this pattern of growth to the presence of two different *β*-galactosidases. However,* B. adolescentis *showed the highest growth rate, followed by* B. breve and B. bifidum. *Meanwhile,* B. infantis *and* B. longum* were the lowest at 65 hr of incubation. Moreover, the differences in growth rate among species of* Bifidobacterium* spp. correlated to different levels of tolerance to aerobic conditions.

### 4.2. Resistance of* Bifidobacterium *spp. to Bile Salts in MRSL Incubated at 37°C

Bile tolerance is one of the most crucial properties as it determines the ability of bacteria to survive in the small intestine and play their functional role as probiotics. A concentration of 0.3% of bile salts closely appropriates the bile level, which are found in the gastrointestinal tract [22]. Common observations among this comparison of different cultures for bile salts tolerance were shown in this study. The highest and lowest resistance of five* Bifidobacterium* spp. were observed in Figure 4. It was shown that* B. infantis *and* B. bifidum* were more resistant to bile salts than the other three species that they reached O.D_660_ of 0.82 and 0.61 at 24 hr, respectively. On the contrary,* B adolescentis *had a dramatically decreased O.D_660_ of 0.31 at 24 hr according to these results. Finally, we summarized that the growths of* Bifidobacterium* spp. were harmed by bile salts. Moreover, these results were in convergence with [23] who reported the tolerance of* Bifidobacterium* to bile or acid. Therefore,* B. infantis *had the highest survival rates followed by* B. bifidum, B. breve,* and* B. longum*, when exposed to bile salts at concentrations ranging from zero to 3 g/L.

**Figure 2:**
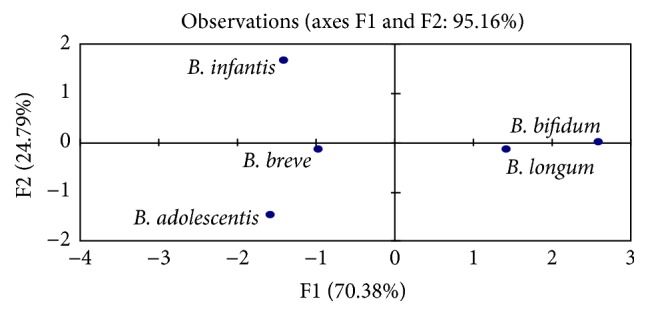
Plots of the *x*-loadings of* Bifidobacterium *spp.

**Figure 3:**
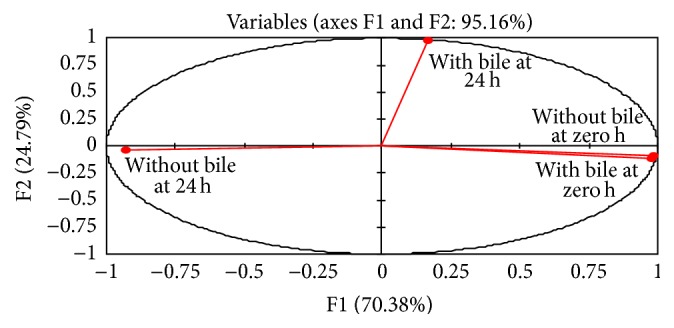
Plots of the scores of* Bifidobacterium *spp.

The result of the PCA was used to study the resistance of* Bifidobacterium *spp. to bile salts. Figures 2 and 3 presented the plots of the scores and the correlation loadings, respectively. The score plots of PCA illustrated the large variability of the five* Bifidobacterium* spp. based on their resistance to bile salts. The loadings are the coefficients of the original variables that define each principal component. Inertia percentage and correlated variables for axes 1 and 2 were displayed in Table 1. Axis 1 explained 70.38% of the total inertia. Axis 2 explained 24.79% of the inertia. Plots of the scores in Figure 2 indicated that the data cloud was mainly bidimensional with respect to the explanatory variables.  Figure 3 showed three clusters of* Bifidobacterium* spp. First cluster included the* B. breve* and* B adolescentis *species. Second cluster included the* B. bifidum *and* B. longum* species. The third cluster (*B. infantis *species) was individualized.

### 4.3. Adhesion of* Bifidobacterium* spp. to Intestinal Epithelial Cells

Major considerations in the choice of* Bifidobacterium *spp. to be used as dietary adjuncts are not only the capability of survival and passing the harmful GI conditions, but also being established within the digestive tract. Caco-2 cells are human intestinal cell lines expressing morphologic and physiologic characteristics of normal human enterocytes [24]. That has been exploited to select and assess probiotics based on their adhesion properties.

Therefore, the adhesion of* Bifidobacterium* spp. to columnar epithelial cells of the small intestine of rabbit was tested as shown in Figure 5. It appeared that the ability of adhesion by* B. adolescentis *to Caco-2 cells was stronger than that of other tested strains, but mainly with resistance to bile salts. In contrary,* B. infantis* was less capable of adhering to epithelial cells and acid production, but it was the best strain resistant to bile salts.

**Figure 4:**
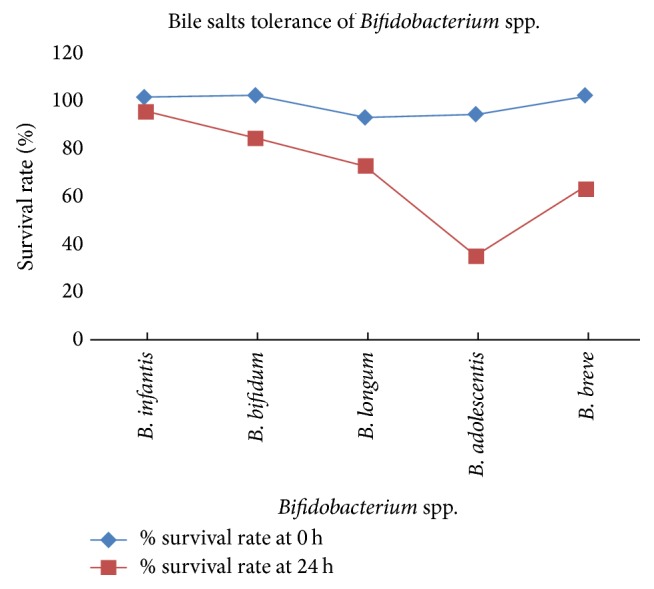
Resistance percentage of* Bifidobacterium* spp. to bile salts.

**Figure 5:**
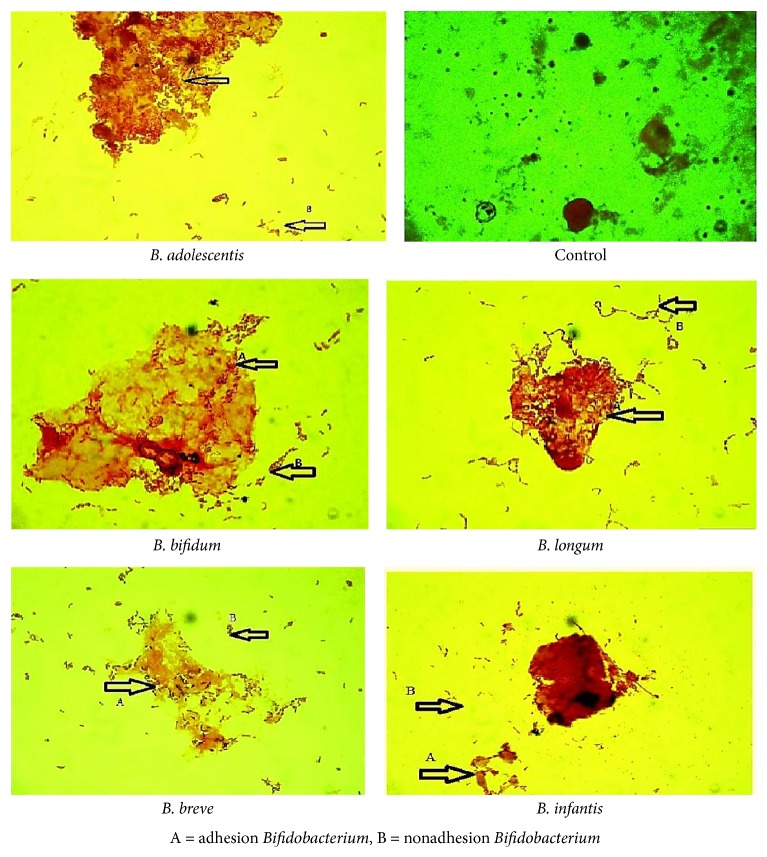
Adhesion of* Bifidobacterium* spp. to intestinal epithelial cells.

According to data shown in Figures 1(a), 1(b), 4, and 5* B. adolescentis *have the highest percentage in survival rate at low pH and stronger adhesion to the epithelial cells. Meanwhile,* B. infantis *is best strain in resistance of bile salts. Therefore, we have chosen these strains to manufacture frozen yogurt.

### 4.4. Physicochemical Characteristics of Frozen Yogurts during 60 Days of Storage at −18°C Acidity and pH

These studies were conducted to see changes in acidity, pH, and total solids of frozen yogurt made with yogurt culture and* Bifidobacterium* spp. during 60 days of storage at −18°C.

Results indicate that there are similar changes of titratable acidity and pH values development observed in different frozen yogurt treated. Only slight changes were found in mix (C + A + B), where the acidity was increased to reach 0.45 at 60 days in the end of storage period. Furthermore, acidity development and pH were steady for five treatments of 60 days of storage. No significant differences (*p* > 0.05) in titratable acidity and pH values were noted among different frozen yogurt mixes during storage periods. These results indicated that the addition of* Bifidobacterium* had no obvious changes.

**Figure 6:**
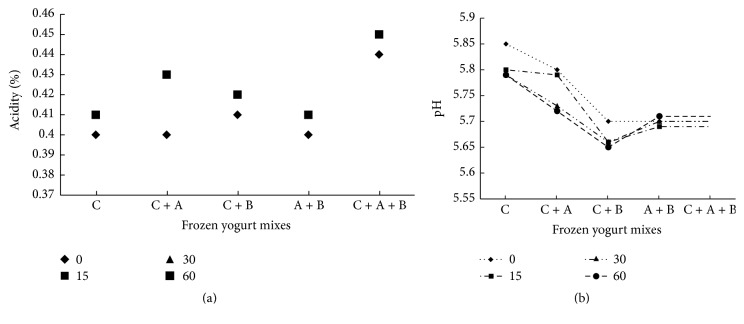
(a) Changes in titratable acidity of frozen yogurt during storage of 0, 15, 30, and 60 d at −18°C. (b) Changes in pH of frozen yogurt during storage of 0, 15, 30, and 60 d at −18°C.

Data in Figures 6(a) and 6(b) were in conformity with the results obtained by [25], who found that titratable acidity of fresh frozen yogurt made with yogurt culture or* Bifidobacterium* spp. culture was 0.45. These indicated that there were no biochemical activities by yogurt culture during storage of the product at −20°C. On the contrary, these results were in disagreement with findings by [26] who reported that the addition of the* Bifidobacterium *spp. led to lower pH.

### 4.5. Total Solids

Total solids play an important role in the quality of frozen yogurt. Results of frozen yogurt samples made with yogurt culture and* Bifidobacterium* spp. during 60 days of storage at −18°C indicated that total solids in all treatments made with yogurt culture and* Bifidobacterium* spp. were about 25.54 to 26.10. These results demonstrated that there was no high significance at* p* < 0.05, among frozen yogurt samples during the storage periods. Therefore, these have close conformities with results obtained by [27], who found that total solids of frozen yogurt made with yogurt culture and* Bifidobacterium* spp. culture with up to 5 weeks of storage at −25°C did not significantly changed. Moreover, [28] reported that a slight increase in total solids was found in all samples during storage period up to 60 days. They attributed an increase to the partial losses in free water during storage.

### 4.6. Changes in Rheological Properties of Frozen Yogurt Made with Yogurt Culture and* Bifidobacterium* spp. during 60 Days of Storage at −18°C

#### 4.6.1. Changes in Hardness (g) of Frozen Yogurt during 60 Days of Storage at −18°C

As seen in Table 2 hardness of frozen yogurt made with yogurt culture only (C) was 75–85 while hardness of frozen yogurt made with* Bifidobacterium* spp. (C + A), (C + B), and (A + B) was 95, 90, and 88, respectively, at 60 days of storage. On the contrary, hardness of mix (C + A + B) was the highest values; it was 85 at fresh samples and 88.33, 96, and 98 for 15, 30, and 60 days' storage period, respectively. These obtained results were in agreement with results obtained by [29] who reported that no significant (*p *< 0.05) differences in hardness were found between frozen yogurts samples. Therefore, the addition of* Bifidobacterium* spp. did not affect the texture of the frozen yogurt.

#### 4.6.2. Changes in Meltdown/Min of Frozen Yogurt during 60 Days of Storage at −18°C

Results in Table 2 showed that the meltdown of frozen yogurt made of yogurt culture (C) was in the range from 116.3 to 265.1 min of fresh to 60 days' storage at −18°C, while the time for collection was increased in mixed yogurt culture +* B. adolescentis* (C + A) from 113.1 to 124.2 of fresh to 60 days' storage at −18°C. Moreover, frozen yogurt made with yogurt culture +* B. infantis * (C + B) was slightly decreased from 138.1 to 124.3. In addition,* Bifidobacterium* spp. culture (A + B) mix was in the range from 286.3 to 275.9 min of fresh to 60 days' storage at −18°C. Therefore, frozen yogurt made with three combinations of cultures (C + A + B) had dramatically increased from 107.2 to 130 from fresh to 60 days of storage at −18°C. Finally, we summarized that only slight changes were found in mix (C + A + B) which increased in meltdown/min of frozen yogurt. Moreover, it was clear that there was no significant (*p* > 0.05) difference in melting time and overrun values between different frozen yogurt mixes. The melting behavior of the product coincided with previous reports focusing on the melting behavior of ice cream with and without probiotics [30]. These findings were in close agreement with the findings of [31].

#### 4.6.3. Changes in Overrun Percentage of Frozen Yogurt Made with Different* Bifidobacterium* spp. 

Overrun is one of the most important quality parameters of frozen desserts, since it affects the texture and consequently the price of the products. Results in Table 2 showed that the overrun levels of the five studied frozen yogurt formulations were low (42.5%–44.50%) and these results were in contrast to [32], who reported that the addition of* Bifidobacterium* spp. led to no high changes in the overrun levels (*p* < 0.05). We hypothesized that it would lead to a poorer foaming capacity and decrease air incorporation in samples with blending components.

#### 4.6.4. Changes in the Viability of* Bifidobacterium* spp. in Frozen Yogurt during 60 Days of Storage at −18°C

**Figure 7:**
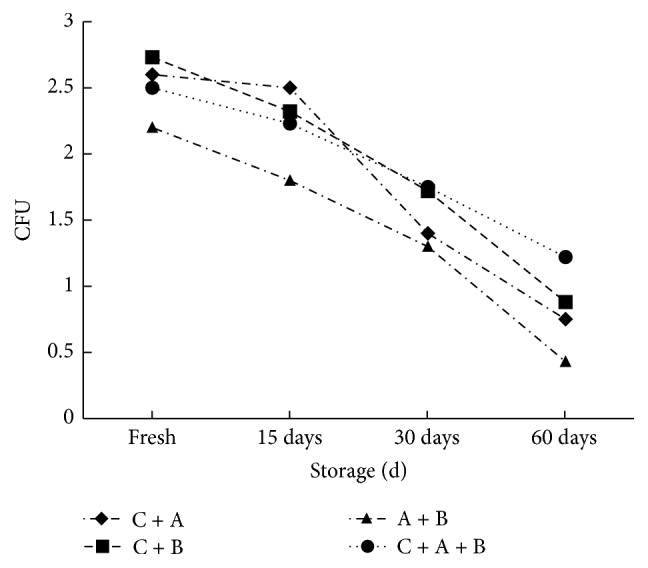
Changes in viability of* Bifidobacterium* spp. in frozen yogurt.

Results in Figure 7 showed the revealed count of* Bifidobacterium* spp. at −18°C decreased with storage period. The count of* Bifidobacterium* spp. for frozen yogurt made with yogurt culture and* B. adolescentis* (C + A) was from 2.6 × 10^8^ to 0.75 × 10^8^ CFU with decrease percent 71.20% from fresh to 60 days of storage period, while that of frozen yogurt made with yogurt culture and* B. infantis *(C + B) was from 2.73 × 10^8^ to 0.88 × 10^8^ CFU with decrease percent 76.80% from fresh to 60 days of storage. Moreover, frozen yogurt made with* Bifidobacterium* spp. culture of* B. adolescentis* +* B. infantis * (A + B) had count of 2.2 × 10^8^ to 0.43 × 10^8^ CFU with decrease percent 80.50% from fresh to 60 days of storage period. Finally, the count of* Bifidobacterium* spp. for frozen yogurt mix made with yogurt culture +* B. adolescentis* +* B. infantis *(C + A + B) was from 2.5 × 10^8^ to 1.22 × 10^8^ CFU with decrease percent 51.20% from fresh to 60 days of storage period. These data were in close agreement with data obtained by [30, 33] who reported that no significant (*p* > 0.05) difference was observed in the count of yogurt bacteria as well as* B. bifidum count* between different frozen yogurt mixes.

### 4.7. Sensory Evaluation of Frozen Yogurt after 60 Days of Storage at −18°C

**Figure 8:**
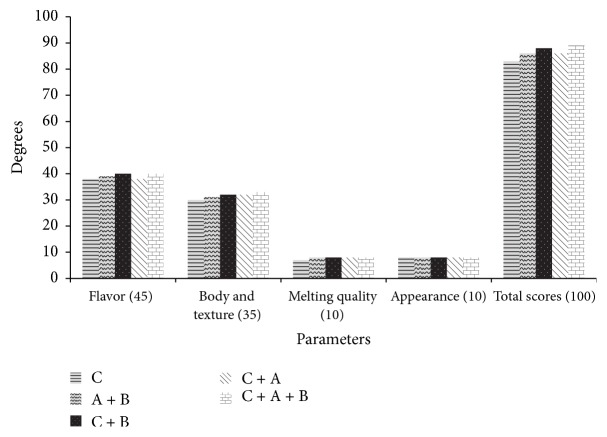
Sensory evaluation of frozen yogurt.

Results in Figure 8 have shown the evaluation scores of frozen yogurt made with yogurt culture and* Bifidobacterium* spp. after 60 days of storage at −18°C. It indicated that there were no high differences between samples in sensory evaluation. It appeared that frozen yogurt made with yogurt culture +* Bifidobacterium adolescentis *+* Bifidobacterium infantis *(C + A + B) gained a high score of 89. In addition, samples made with yogurt culture +* Bifidobacterium infantis *(C + B) gained score of 88 in total as well.

**Figure 9:**
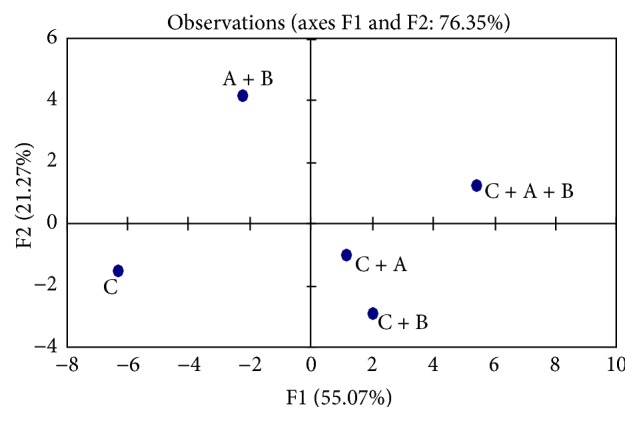
Plots of the *x*-loadings.

There are at least two important aspects that should be highlighted while analyzing frozen yogurt. First, consumers are used to the flavor of dairy products produced with traditional yogurt bacteria, which would lead to lower sensory scores to products that do not fit into this category. Secondly,* Bifidobacterium* spp. are heterofermentative organisms, which are able to produce several types of organic acids (lactic, acetic, and formic acid) and ethanol [34] which can induce important flavor modifications. Considering the potential benefits provided by the probiotic microorganisms, process adjustments could be implemented in order to overcome any possible flavor or aroma issues. In spite of the slightly acidic flavor of their samples, unfamiliar to our consumers, all these samples were acceptable. The results in Figure 9 closely agreed with results obtained by [35], who found that the overall acceptance of probiotic ice cream depends on the preferred and accepted pH.

Results of the PCA were used to analyze physicochemical characteristics, some rheological properties, and sensory evaluation of frozen yogurts. Figure 9 presented the correlation loadings. The scores plot of PCA illustrated the large variability of five mixes of frozen yogurt based on different species of* Bifidobacterium* spp. during 60 days of storage at −18°C. Loadings were the coefficients of the original variables of each principal component. Inertia percentage and correlated variables for axes 1 and 2 were displayed in Table 3. Axis 1 explained 55.08% of the total inertia. Axis 2 explained 21.27% of the inertia. With respect to the explanatory variables, Figure 9 shows four clusters of mixes. The first cluster included the C + A and C + B, whereas the second, third, and fourth clusters were C, A + B, and C + A + B, respectively, individualized.

## 5. Conclusion

*Bifidobacterium* spp. can grow well and have ability to withstand different conditions of acidity and bile. Moreover, frozen yogurt can serve as an excellent vehicle for dietary incorporation of probiotic bacteria. On the contrary, frozen storage of the products has little effects on the survival of* Bifidobacterium* spp., which are sufficient to offer the suggested therapeutic effects. Supplementation with* Bifidobacterium* spp. has been found to exert a little effect on flavor or compositional characteristics of frozen yogurt. Our previous study indicated that there were no significant difference changes (*p* > 0.05) during adding different* Bifidobacterium *spp. in the physiochemistry or sensory evaluation of frozen yogurt.

## Figures and Tables

**Table 1:** Discriminate variables factors of principal components analysis to study the resistance of *Bifidobacterium* spp. to bile salts.

	F1	F2
Proper value	2.82	0.99
Variability (%)	70.38	24.79
Cumulative (%)	70.38	95.17

**Table 2:** Changes in some rheological properties of frozen yogurt made with yogurt culture and *Bifidobacterium* spp. culture during 60 days of storage −18°C.

Treatment	Storage time/days	Hardness/g	Meltdown/min	% Overrun
C	0	75 ± 1^j^	116.3 ± 1^o^	24.5
	15	78.58 ± 0.1^hi^	194.8 ± 1^e^	
	30	80 ± 1^h^	188 ± 1^f^	
	60	85 ± 1^f^	265.1 ± 1^d^	

C + A	0	82.07 ± 1^g^	113.1 ± 1^p^	42.86
	15	87 ± 1^e^	135 ± 1^h^	
	30	92 ± 1^c^	128 ± 1^j^	
	60	95 ± 1^b^	124.2 ± 1^l^	

C + B	0	78 ± 1^i^	138.1 ± 1^g^	43
	15	80 ± 1^h^	120.5 ± 1^m^	
	30	87 ± 1^e^	118 ± 1^n^	
	60	90 ± 1^d^	124.3 ± 1^l^	

A + B	0	76 ± 1^j^	286.3 ± 1^b^	43.7
	15	79 ± 1^hi^	323 ± 1^a^	
	30	83 ± 1^g^	276 ± 1^c^	
	60	88 ± 1^e^	275.9 ± 1^c^	

C + A + B	0	85.05 ± 1^f^	107.2 ± 1^q^	44.5
	15	88.33 ± 1.26^e^	126 ± 1^k^	
	30	96 ± 1^b^	120.1 ± 1^m^	
	60	98 ± 1^a^	130 ± 1^i^	

Values are the average of three individual samples each analyzed in duplicate ± standard deviation. Different lowercase superscript letters, respectively, indicate significant difference (*p* < 0.05) analyzed by Duncan's multiple range test.

**Table 3:** Discriminate variable factors of principal components analyses of analyzed physicochemical characteristics, some rheological properties, and sensory evaluation.

	F1	F2
Proper value	15.97	6.17
Variability (%)	55.08	21.27
Cumulative (%)	55.08	76.35
